# Experimental Study of 5-fluorouracil Encapsulated Ethosomes Combined with CO2 Fractional Laser to Treat Hypertrophic Scar

**DOI:** 10.1186/s11671-017-2425-x

**Published:** 2018-01-18

**Authors:** Zhen Zhang, Jun Chen, Jun Huang, Yan Wo, Yixin Zhang, Xiangdong Chen

**Affiliations:** 1 Department of Dermatology, Shanghai Ninth People’s Hospital, Shanghai JiaoTong University School of Medicine, Shanghai, 200011 China; 2 Department of Laser and Aesthetic Medicine, Shanghai Ninth People’s Hospital, Shanghai JiaoTong University School of Medicine, Shanghai, 200011 China; 3 Department of Dermatology, The Chongqing Hospital of Traditional Chinese Medicine, Chongqing, China; 4 Department of Human Anatomy, Histology and Embryology, School of Medicine, Shanghai Jiao Tong University, Shanghai, 200025 China; 5 Department of Plastic and Reconstructive Surgery, Shanghai Ninth People’s Hospital, Shanghai JiaoTong University School of Medicine, Shanghai, 200011 China

**Keywords:** CO_2_ fractional laser, Hypertrophic scar, 5-Fluorouracil, Ethosomes, Transdermal delivery

## Abstract

**Objective:**

This study is designed to explore permeability of ethosomes encapsulated with 5-florouracil (5-FU) mediated by CO_2_ fractional laser on hypertrophic scar tissues. Moreover, therapeutic and duration effect of CO_2_ fractional laser combined with 5-FU encapsulated ethosomes in rabbit ear hypertrophic scar model will be evaluated.

**Methods:**

The permeated amount of 5-FU and retention contents of 5-FU were both determined by high-performance liquid chromatography (HPLC). Fluorescence intensities of ethosomes encapsulated with 5-FU (5E) labeled with Rodanmin 6GO (Rho) were measured by confocal laser scanning microscopy (CLSM). The permeability promotion of 5E labeled with Rho in rabbit ear hypertrophic scar mediated by CO_2_ fractional laser was evaluated at 0 h, 6 h, 12 h, 24 h, 3 days and 7 days after the irradiation. The opening rates of the micro-channels were calculated according to CLSM. The therapeutic effect of 5EL was evaluated on rabbit ear hypertrophic scar in vivo. Relative thickness of rabbit ear hypertrophic scar before and after the treatment was measured by caliper method. Scar elevation index (SEI) of rabbit ear hypertrophic scar was measured using H&E staining.

**Results:**

The data showed that the penetration amount of 5EL group was higher than 5E group (4.15 ± 2.22 vs. 0.73 ± 0.33; *p* < 0.05) after 1-h treatment. Additionally, the penetration amount of 5EL was higher than that of the 5E group (107.61 ± 13.27 vs. 20.73 ± 3.77; *p* < 0.05) after 24-h treatment. The retention contents of the 5EL group also showed higher level than 5E group (24.42 ± 4.37 vs.12.25 ± 1.64; *p* < 0.05). The fluorescence intensity of Rho in hypertrophic scar tissues of the 5EL group was higher than that of the 5E group at different time points (1, 6, and 24 h). The opening rates of the micro-channels were decreased gradually within 24 h, and micro-channels were closed completely 3 days after the irradiation by CO_2_ fractional laser. The relative thickness and SEI of rabbit ear hypertrophic scar after 7 days of treatment in the 5EL group were significantly lower than the 5E group.

**Conclusion:**

CO_2_ fractional laser combined with topical 5E can be effective in the treatment of hypertrophic scar in vivo and supply a novel therapy method for human hypertrophic scar.

## Background

Hypertrophic scar is a cutaneous condition characterized by deposits of excessive amounts of collagen which gives rise to a raised scar, but not to reach the observed degree of keloids [1]. A total of 100 million patients developed scars in developed countries every year, as a result of 55 million elective operations and 25 million operations after trauma [2]. After surgical operation, about 60% of patients developed hypertrophic scars postoperatively, typically during the first 3 months. Most hypertrophic scars are still hypertrophic in 12 months after surgical operations [3]. Currently, the common treatment methods include surgical and non-surgical treatments. Pressure, radiation therapy, chemotherapy, and other medication surgical treatments are the major therapy treatment. So far, these therapies could not achieve the desired therapeutic effect due to their defects [4, 5]. Drug therapy is one of the most common non-surgical treatments for pathological scar to external painting based on local injections. Due to drug side effects, local injections are often limited to small-scale and low-dose treatment. Moreover, because of short drug half-life, local drug injections always failed to maintain long-lasting high concentrations on scar; therefore, repeated injections are often needed [6]. Considering density of scar tissue and severe pain, injection is not generally acceptable for patients. Despite of the advantages including painless, no side effect to the liver, and convenient for long-term external usage [7, 8], drugs could not enter the scar due to special organizational structure of pathological scars. Recent reports also indicated that topical medication is difficult to penetrate the scar tissue [9]. So the external usage for drug is limited currently.

Ethosomes are a novel lipid carrier used by Touitou in 2000 [10], effective on delivering drugs through the skin. The main component of ethosomes is ethanol, which can change the tight arrangement of cuticle lipid molecules, enhance lipid fluidity, and promote ethanol liposome membrane flexibility and mobility. Ethanol from ethosomes can also accelerate the deformation of the stratum corneum and enhance its carrying and penetration capability of drug through disordered stratum corneum in the skin [11–13]. However, being different with normal skin tissue, scar tissue has special structure. In our previous study, we demonstrated that ethosomes are a highly efficient carrier for drug in human scar [14]. It is indispensable that the drug in scar tissue is not well-distributed, but being significantly decreased from the outside to the inside of the skin. Drug is mainly accumulated in the epidermis and superficial layer of the dermis but not in all layers of the dermis, which decreases the anti-scarring effect.

Ablative fractional laser therapy, a cosmetic technology, is very popular and widely accepted modality currently and mainly used for clinical treatment of facial wrinkles, superficial scars, pigmentation disorders, and improving skin texture [15–17]. Based on dot matrix photothermolysis theory [18], ablative fractional laser produces an array-like arrangement of tiny laser beam and disrupts the skin barrier by creating ablative microscopic vertical channels surrounded by a zone of thermal damage, inducing a wound healing response to moderate skin appearance [19–22]. It has been reported that the main barrier of drug percutaneous absorption is the stratum corneum [23, 24]. Recent studies have shown that, one of the most commonly used clinical ablative fractional laser, CO_2_ fractional laser can breakdown stratum corneum and form dense microporous channels, thereby undermining the main barrier of impeding the drug percutaneous absorption and giving good transdermal drug penetration promoting prospects [25–28].

In this study, we explored CO_2_ fractional laser-mediated ethosomal gel carrying anti-scarring drug 5-florouracil (5-FU) in human scar samples and rabbit ear hypertrophic scar model. Using this new approach, we focused on improving the efficiency of anti-scarring drug administration in order to shorten the long scar treatment process. For the first time, rabbit ear hypertrophic scar model has been applied to determine CO_2_ fractional laser-mediated nanoscale ethosomes carrying 5-FU on hypertrophic scars and drug penetration effect. Together, it has been suggested that CO_2_ fractional laser combined with topical drug supplies a novel therapy method for human hypertrophic scar.

## Methods

### 5-FU Encapsulated Ethosomes

Touitou’s method [10] was used as preparation for 5-FU encapsulated ethosomes. Particle size distribution of 25 °C and polydispersity index (PDI) were determined using laser particle size analyzer (*λ* = 632.8 nm). PDI < 0.1 indicates the even particle size distribution, and PDI > 0.3 suggests inhomogeneous particle size distribution. Transmission electron microscopy (TEM) was used for the observation of the morphology of ethosomes. Ultracentrifugation method [29] was used for measuring the encapsulation efficiency (EE) of ethosomes in this study.

### High Performance Liquid Chromatography

The concentration of 5-FU in the receptor compartment of the Franz cells was determined by a Waters 2695 HPLC system (Meadows Instrumentation, Illinois) and 2487 ultraviolet detector with a reverse-phase Diamonsil TM C18 column (250 mm × 4.6 mm, 5 μm). 5-FU was detected at 265 nm with a mobile phase of methanol–H_2_O (5:95 *v*/*v*) at a flow rate of 1 ml/min. The data were analyzed by peak area and the external standard method, using a Waters Empower System.

### Confocal Laser Scanning Microscopy

Human hypertrophic scar tissue was analyzed by confocal laser scanning microscopy (CLSM) at 10 μm increments. Fluorescence intensity was analyzed using a Laser Scanning Microscope LSM 510 (Zeiss, Jena, Germany) with a Fluar 10×, 0.5 numerical aperture objective lens. Optical excitation was carried out with a 543-nm HeNe laser, and fluorescence emission was detected above 560 nm for rhodamine 6GO (Rho). Images were analyzed by Release Version 4.0 SP2 image analysis software to calculate fluorescence distribution and fluorescence intensity of scar tissues. In 10 × 10 times the field of view, the size and distribution of the pixel values was to determine 5-FU EG (labeled with Rho) penetration range and by semi-quantitative method.

### 5-FU Ethosomes Permeability Assay in Human Scar Tissue

Human hypertrophic scar specimens from the Shanghai Jiaotong University Affiliated Ninth People’s Hospital of Plastic Surgery are from scar excision in patients with three cases, all women, aged 21–35 years; the scar is located on the shoulder and back; hypertrophic scar course of 6 months to 1 year; scar tissue integrity; no ulceration; no infection lesions; and the lowest scar must also be higher than the skin, and the patient has not received any treatment since the illness. General anesthesia was used to eliminate the effect of local anesthetics on scar tissue. The obtained specimens were immediately removed from the subcutaneous tissue, protected the epidermis, made into a thickness of 4.0 mm, an area of 3 cm × 3 cm scar tissue samples, washed with saline at room temperature, dried with gauze dry surface moisture, wrapped with aluminum foil, and stored in a refrigerator at − 20 °C. Human hypertrophic scar tissue was removed and stored in − 20 °C freezer before use. Human hypertrophic scar tissue was immersed in pH 7.4 PBS at 25 °C for 2 h, and the water on tissue surface was removed gently with dry gauze. All epidermal tissue regions of hypertrophic scar tissue were CO_2_ fractional laser irradiated. Laser parameters are followed immediately by DeepFX mode, 25 mj energy density, 20% coverage, 300 Hz emission frequency, on the 10th spot size, and without overlap. All irradiated samples were collected by using HPLC detection, to determine the scar tissue content of 5-FU. Histopathological sections are immediately used for CLSM assay.

### Rabbit Ear Hypertrophic Scar Model

The method of rabbit ear hypertrophic scar model was described briefly below: 12 New Zealand white rabbits, male, and weighing 2 kg (SLAC, Shanghai) were housed separately for 2 weeks. For rabbit anesthesia, lumianning was mixed with 1.5 mg ketamine per 100 g weight via intramuscular injection. All the wounds were surrounded by the central point of the rabbit ears. Each round wound with a diameter of 1 cm was defective, and then the perichondrium was removed. Twenty-eight days after surgery, the rabbit ear scab off the wound and healed completely, with a visible diameter of about 0.9 cm hypertrophic scars. The scar was bright red, bumps were on the skin around the obvious hyperplasia, and the scar was thick and hard.

### Micro-channel Opening Rates Determination

Different time points after CO_2_ fractional laser treatment in rabbit ear hypertrophic scar model, red fluorescence Rodanmin 6GO with ethosomes was applied to scar tissues. Three hours later, CLSM was used for determination of channel opening rate. Channel opening rate = open channel number/(open channel number + closed channel number) × 100%. The percentage of the channel opening rate reflects the effect of CO_2_ fractional laser penetration effectiveness.

### H&E Staining

Generally, rabbits are sacrificed using 10% pentobarbital i.v. injection (dose of 5 times of normal dose for anesthesia, 35 mg/kg). Immediately remove the specimens and proceed to H&E staining step. Hematoxylin and eosin staining method is according to the standard H&E staining protocol.

### Scar Elevation Index and Relative Thickness Determination

Twenty-eight days after induction of rabbit ear hypertrophic scars, four groups of intervention are performed: 5EL group: enthosomal gels encapsulated with 5-FU combined with CO_2_ fractional laser; 5E group: the enthosomal gels encapsulated with 5-FU; CO_2_ group: CO_2_ fractional laser treatment; and control group. We defined A as thickness of the thickest part of rabbit ear hypertrophic scar tissue, and B as thickness of 1.0 cm away from the thickest part of rabbit ear hypertrophic scar tissue (close to the center point). The relative thickness is calculated by A/B. Dermal scar hypertrophy was illustrated by the SEI. SEI = the area of newly formed dermis/the area of unwounded dermis. SEI > 1.5 depicts a hypertrophic scar.

### Statics

All experiments were repeated for three times. Data are presented as mean ± standard deviation (SD). Statistical analysis was performed using SPSS version 19.0 (SPSS Inc., Chicago, USA). Student’s *t* test was used to calculate the significance. *p value < 0.05 was considered statistically significant; **p value < 0.01 was considered statistically very significant; ***p value < 0.001 was considered statistically extremely significant, and ****p value < 0.0001 was considered statistically highly extremely significant.

## Results

### Quality Assessment of Ethosomes Encapsulated with 5-florouracil(5E)

**Fig. 1:**
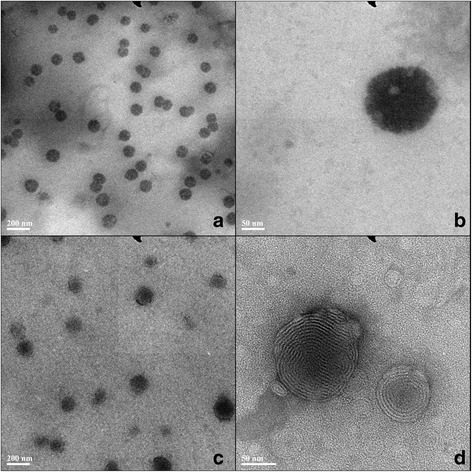
Transmission electron microscope images of 5-FU ethosomes. Ethosomes of solutions (**a** and **b**) and ethosomes of gel (**c** and **d**)

First of all, to validate the quality of ethosomal gel encapsulated with 5-florouracil (5E), the morphology and diameters were detected by transmission electron microscope (TEM) observation. Ethosomes in suspension state formed a universal-sized complete circle or oval-spherical vesicle, which is less than 100 nm under a microscope (Fig. 1a–b). After gel formulation, ethosomes remained intact (Fig. 1c–d). Using laser particle size analyzer, ethosomes in suspension state appear to be with a diameter of 87.72 ± 9.27 nm, and the PDI was 0.10 ± 0.01. In the meanwhile, the particle size of the ethosomes was 98.78 ± 10.88 nm, and PDI was 0.11 ± 0.02. A statics comparison shows that the particle size has no significant difference (*p* > 0.05). Additionally, both PDIs had no significant difference (*p* > 0.05). In addition, the data obtained from laser particle size analyzer and TEM showed the comparable results. Moreover, the entrapment efficacy (EE) was also determined using the ultracentrifugation method (Table 1). The results showed that EE of ethosomal suspension was 10.47 ± 1.47%, and EE of ethosomal gel was 11.56 ± 1.12% (*n* = 6), with no significance (*p* > 0.05). In conclusion, no morphology and particle size changes were found in suspension state and gel state of ethosomes.

**Table 1:** Characterization of 5-FU ES and 5-FU EG

	Particle size (nm)	PDI	EE (%)
5-FU ES (*n* = 6)			
Mean	**87.72**	**0.10**	**11.58**
STD	9.27	0.01	1.12
5-FU EG (*n* = 6)			
Mean	98.7*	0.11**	10.47***
STD	10.88	0.02	1.74

Mean ± SD; *p < 0.05, Particle size of 5- FU EG compared to 5-FU ES using one-way ANOVA; ** *p* < 0.01, PDI of 5- FU EG compared to 5-FU ES using one-way ANOVA; *** *p* < 0.001, EE(%) of 5- FU EG compared to 5-FU ES using one-way ANOVA

### CO_2_ Fractional Laser Promotes 5E Permeability Through Hypertrophic Scars In Vitro

**Fig. 2:**
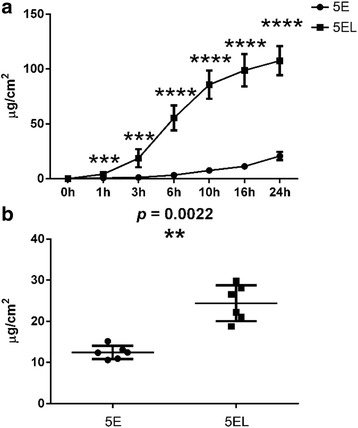
CO_2_ fractional laser promotes 5E permeability through hypertrophic scars in vitro*.* **a** Comparison of the permeation of 5E and 5EL group in a 24-h study of human hypertrophic scar in vitro. Values were expressed as the mean ± SD. *n* = 6. **b** The accumulative retention of 5-FU in hypertrophic scar in vitro after application for 24 h. *n* = 6. ***p value < 0.001 was considered statistically extremely significant, ****p value < 0.0001 was considered statistically highly extremely significant

After evaluation quality of ethosomes encapsulated with 5-florouracil (5-FU), we explored its permeability in human hypertrophic scars with or without CO_2_ fractional laser in vitro*.* Human hypertrophic scar tissue was irradiated by CO_2_ fractional laser and then 5E was evenly applied. The cumulative concentrations of 5-FU were determined at 1, 3, 6, 10, 16, and 24 h by HPLC ( 2). We compared 5-FU cumulative concentrations of CO_2_ fractional laser combined with ethosomes encapsulated with 5-florouracil (5EL) group and 5E group at different time points. At 1 h, 5-FU cumulative concentration of 5EL group was 4.15 ± 2.22 μg/ml/cm^2^, which was higher than the concentration of 5E group (0.73 ± 0.33 μg/ml/cm^2^, *p* < 0.001). In the long-term treatment (24 h), 5-FU cumulative permeation concentration of the 5EL group (107.61 ± 13.27 μg/ml/cm^2^) was also higher than that of the 5E group (20.73 ± 3.77 μg/ml/cm^2^, *p* < 0.0001). In the earlier time points, 3, 6, 10, and 16 h, the 5EL groups always showed higher 5-FU cumulative permeation concentration than the 5E groups (Fig. 2a). Moreover, the retention amount of 5-FU in the 5EL group was 24.42 ± 4.37 μg/cm^2^, which is higher than 5E group (12.45 ± 1.64 μg/cm^2^, *p* < 0.01, *n* = 6) (Fig. 2b). This result suggested that CO_2_ fractional laser irradiation can significantly promote 5-FU contained liposome through human hypertrophic scar tissue and help 5-FU retention in hypertrophic scar tissue in vitro.

**Table 2:** The permeation of 5-FU in human hypertrophic scar in vitro at different time points

*n* = 6	1 h	3 h	6 h	10 h	16 h	24 h
5E						
Mean	**0.73**	**1.21**	**3.3**	**7.54**	**11.34**	**20.73**
STD	0.33	0.33	0.59	1.55	2.03	3.77
5EL						
Mean	**4.15**	**18.64**	**55.41**	**85.66**	**98.8**	**107.61**
STD	2.22	8.24	11.38	12.91	14.75	13.27

mean ± SD; 5EL: the enthosomal gels encapsulated with 5-FU combined with CO_2_ fractional laser, *n* = 6; 5E: the enthosomal gels encapsulated with 5-FU, *n* = 6

**Fig. 3:**
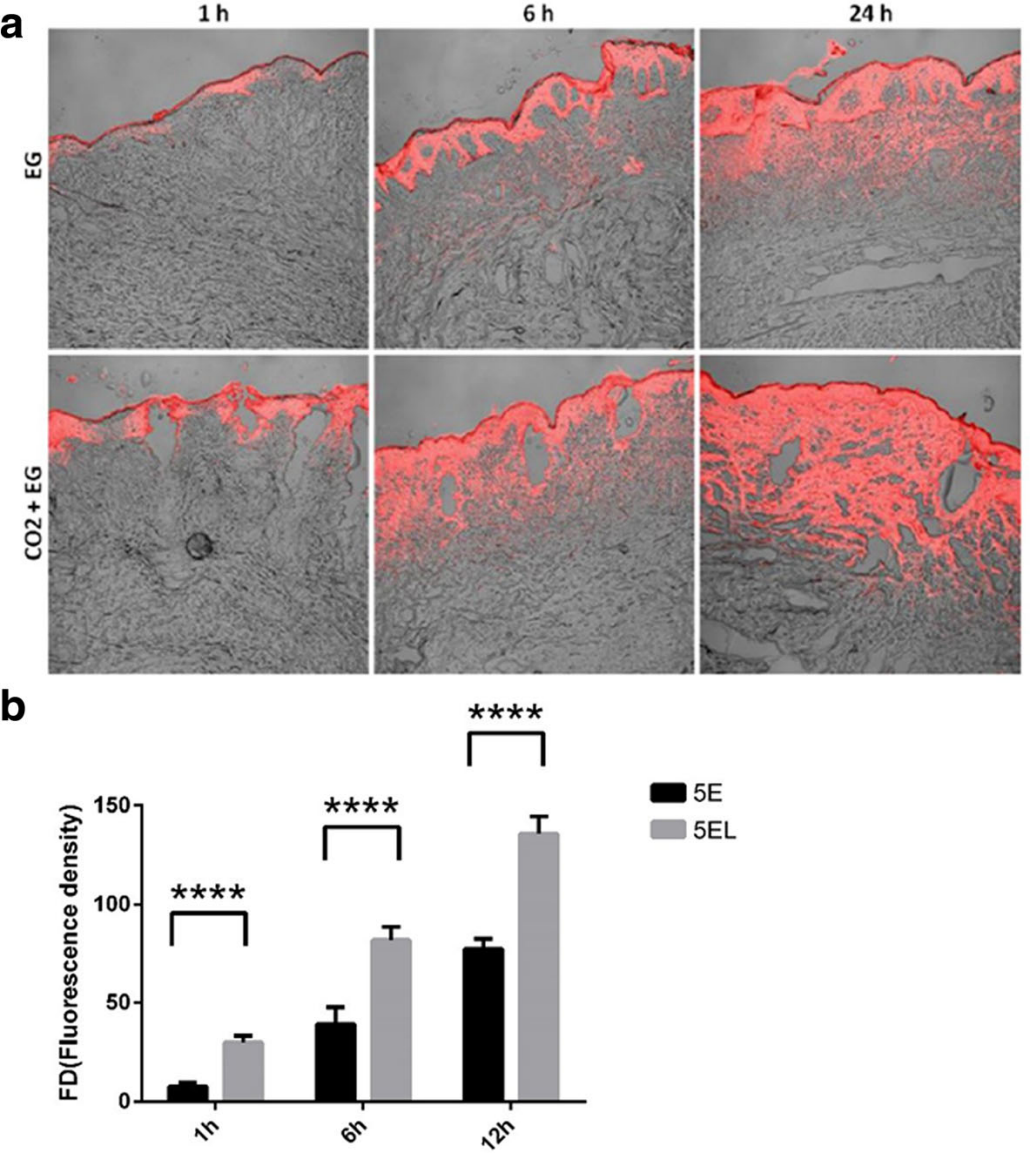
CO_2_ fractional laser promotes permeability through hypertrophic scars in vitro*.* **a** The red fluorescence is labeled ethosomes permeated hypertrophic scar tissues after 1, 6, and 24 h in vitro. **b** The fluorescence intensity of Rhodamine 6GO labeled ethosomes on hypertrophic scar tissue in vitro after 1, 6, and 24 h. ****p value < 0.0001 was considered statistically highly extremely significant

The depth and extent of 5-FU penetration were determined using 5E labeled with Rho to evaluate the enhancing penetration effect of CO_2_ fractional laser in vitro. Fluorescence assay was used to indicate intensity of 5E and 5EL groups at 1, 6, and 24 h after 5-FU treatment (Fig. 3a). The results showed that after 1-h 5EL treatment, Rho fluorescence was distributed in the epidermis and dermis shallow layer, especially around CO_2_ fractional laser-induced gasification zone. In contrast, without CO_2_ fractional laser irradiation, Rho fluorescence distribution was limited to the epidermis area in the 5E group. After 6 h treatment in the 5EL group, Rho fluorescence expanded to the deep dermis and exhibited more accumulation. Although fluorescence distribution of the 5E group started to appear in the dermis, the fluorescence intensity decreased from the dermis to epidermis gradually. After 24 h treatment, fluorescence of two groups was widely distributed in the whole skin tissue, but the intensity of fluorescence in the 5EL group was significantly higher. Further, Release Version 4.0 SP2 image analysis software was used for quantitative analysis to calculate fluorescence intensity for both groups. The quantitative analysis results showed significant increased Rho fluorescence intensity in the 5EL group than the 5E group (1 h: 59.61 ± 6.39 vs.6.39 ± 1.64, *p* < 0.0001; 6 h: 163.32 ± 13.23 vs. 49.89 ± 4.01, *p* < 0.0001; 24 h: 270.36 ± 8.73vs. 148.25 ± 16.89, *p* < 0.0001) (Fig. 3b). In summary, CO_2_ fractional laser irradiation significantly promotes the 5E penetration into the human hypertrophic scar tissue in vitro.

### Duration of CO_2_ Fractional Laser Enhances 5-FU Penetration In Vivo

**Fig. 4:**
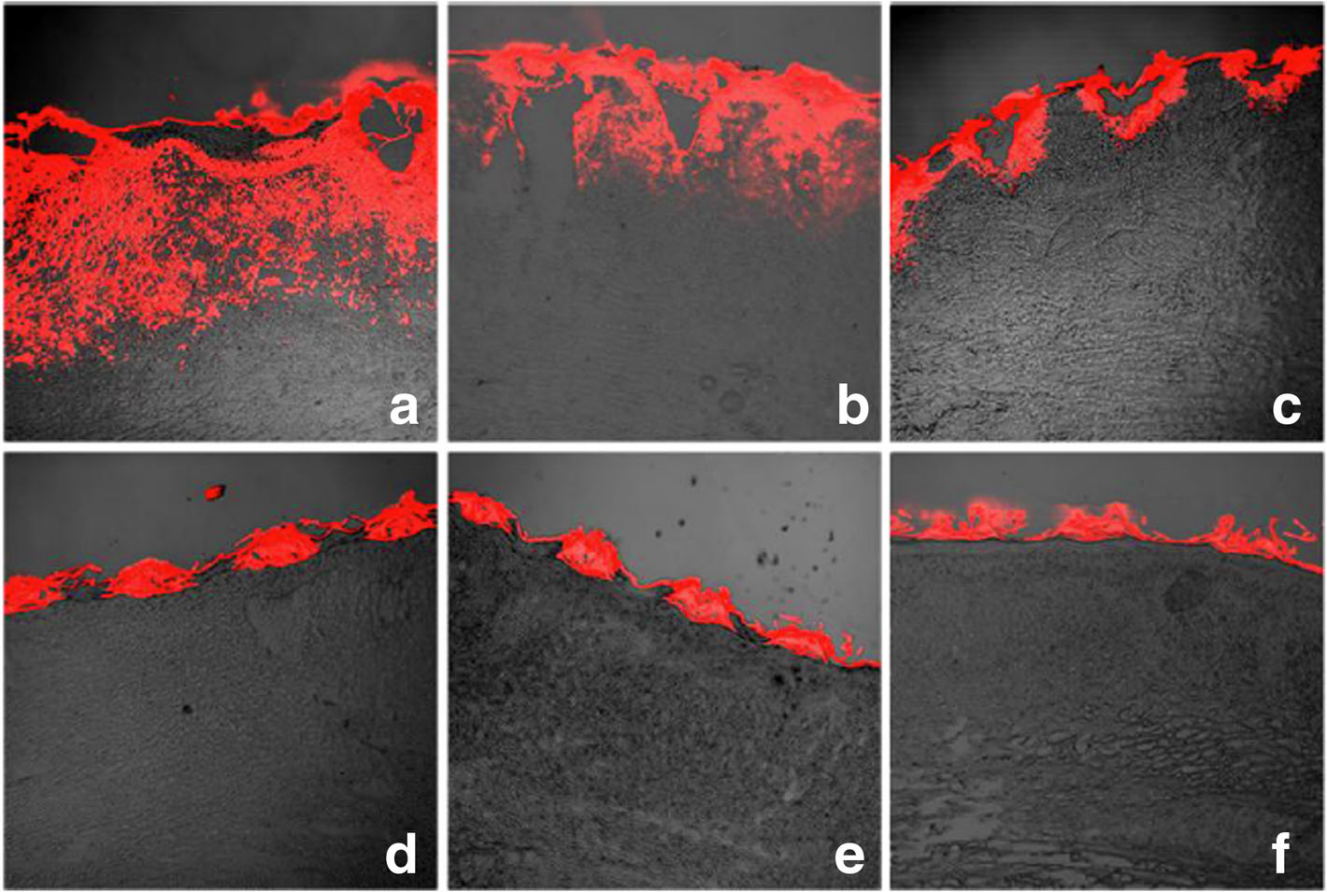
CO_2_ fractional laser promotes micro-channel opening rates in vivo. The red fluorescence is labeled ethosomes permeate rabbit ear hypertrophic scar tissues 0 h (**a**), 6 h (**b**), 12 h (**c**), 24 h (**d**), 3 days (**e**), and 7 days (**f**) after CO_2_ fractional laser treatment in vivo

To obtain more substantial evidence of CO_2_ fractional laser effect, we performed 5-FU penetration in vivo using rabbit hypertrophic scar model. Rabbit hypertrophic scar model setup was described as Method. Different time points (3 h, 6 h, 12 h, 24 h, 3 days, and 7 days) after application of CO_2_ fractional laser, 5E or 5EL treatment was performed and fluorescence distribution was determined immediately by CLSM (Fig. 4). Rho fluorescence was extensively visible and widely distributed in the dermis layer of the rabbit ear hypertrophic scars, which were closer to the ablative zone (Fig. 4a). These results may indicate Rho mixed with 5E primarily infiltrate through the porous channel after CO_2_ fractional laser irradiation. Fluorescence can be detected in the ablative zone surrounding the dermal tissue after 6 h 5EL, even with a small distribution area (Fig. 4b). Fluorescence distribution area continues to shrink 12 h after drug treatment, which indicates that microporous channels are gradually closed when the wound heals (Fig. 4c). After 24 h, 3 days, and 7 days treatment of CO_2_ fractional laser irradiation, fluorescence could only be found within the crust pore around openings and no penetration into the dermis (Fig. 4d–f), which suggests that CO_2_ fractional laser penetration effect has disappeared with complete re-epithelialization of the epidermis. Our data suggested that micro-channel opening plays a critical role in drug penetration with 5EL treatment. Thereafter, we calculated micro-channel opening rates of 3 h, 6 h, 12 h, 24h, 3 days and 7 days after laser irradiation, respectively. At the time points of 3 and 6 h, microporous channels were all open, which indicated micro-channel opening rates were 100%. Micro-channel opening rates started to be decreased to 90.59% at 12 h and 15.58% at 24 h. In particular, microporous channels were all closed at 3 and 7 days after 5EL treatment. Taken together, these results suggest that CO_2_ fractional laser controls drug penetration in rabbit hypertrophic scar tissue in vivo.

### The Therapeutic Effect of 5EL on Hypertrophic Scar In Vivo

**Fig. 5:**
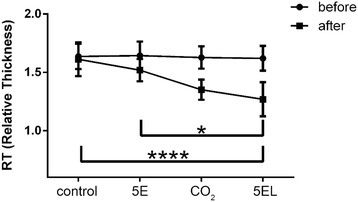
Comparison of relative thickness of rabbit hypertrophic scars before and after applied different treatments. 5EL: the enthosomal gels encapsulated with 5-FU combined with CO_2_ fractional laser, *n* = 14; CO_2_: CO_2_ fractional laser only, *n* = 12; 5E group: the enthosomal gels encapsulated with 5-FU, *n* = 14; control: blank, *n* = 16. *p value < 0.001 was considered significant, ****p value < 0.0001 was considered statistically highly extremely significant

To understand deeply the 5EL treatment, we performed determination of the relative thickness of rabbit ear hypertrophic scar. After rabbit ear hypertrophic scar model setup, relative thickness values of before and after 5EL treatment were calculated. No significant difference was found in before treatment groups. However, the relative thickness of 5EL group was 1.27 ± 0.15, which was significantly lower than 5E group (1.52 ± 0.10, *p* < 0.05) and untreated (1.61 ± 0.15, *p* < 0.0001) group (Fig. 5). Interestingly, there was no significant change between the CO_2_ laser treatment only group and the 5EL group. These data suggest that CO_2_ fractional laser plays a dominant role in curing rabbit ear hypertrophic scar in vivo.

**Fig. 6:**
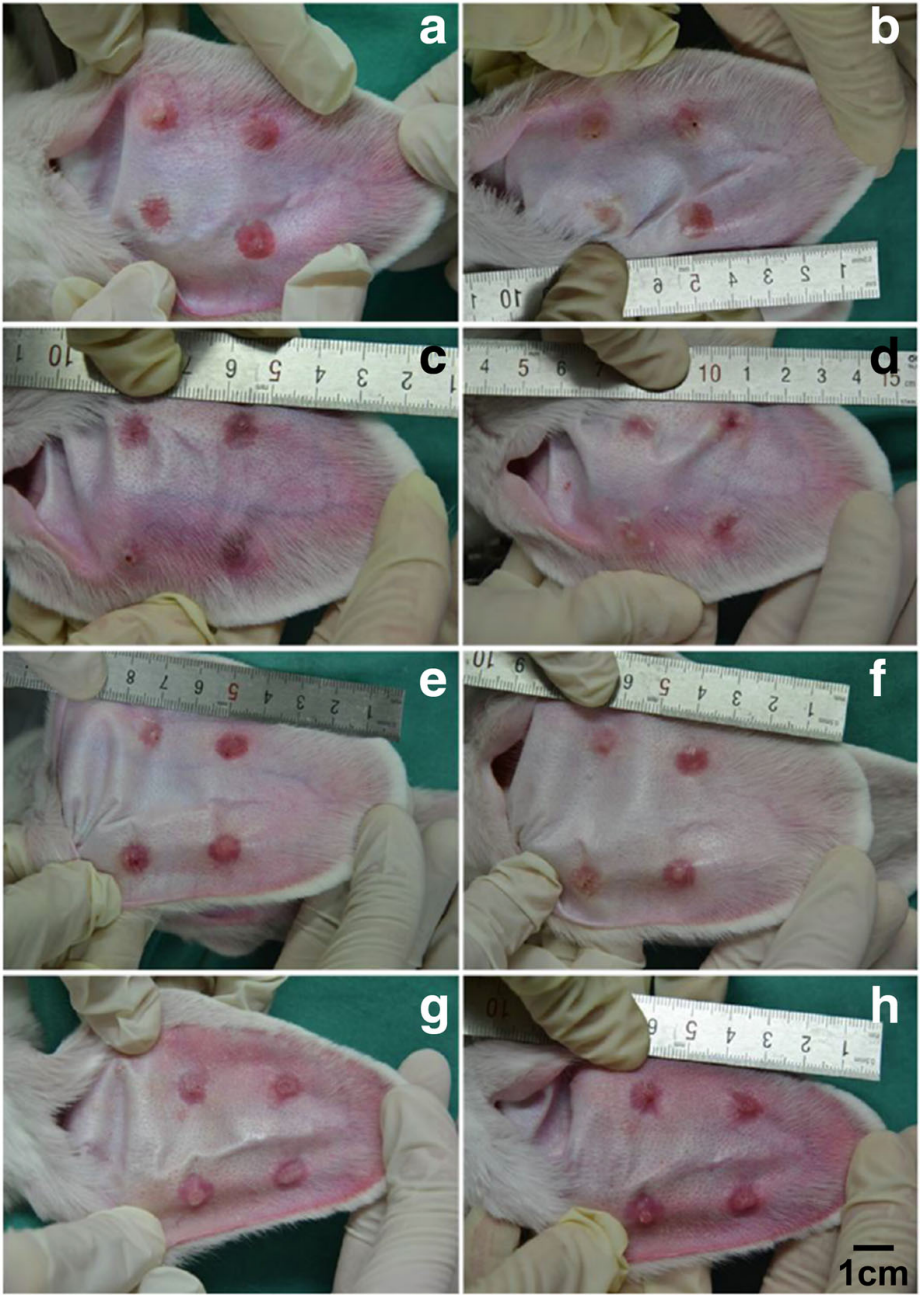
Photographic imaging of rabbit hypertrophic scars before and after applied different treatments for 7 days. The 5EL group: the enthosomal gels encapsulated with 5-FU combined with CO_2_ fractional laser, before (**a**) and after (**b**) treatment for 7 days; CO_2_ group: CO_2_ fractional laser, before (**c**) and after (**d**)treatment for 7 days; 5E group B: the enthosomal gels encapsulated with 5-FU, before (**e**) and after (**f**) treatment for 7 days; and blank group: blank, before (**g**) and after (**h**) treatment for 7 days

**Fig. 7:**
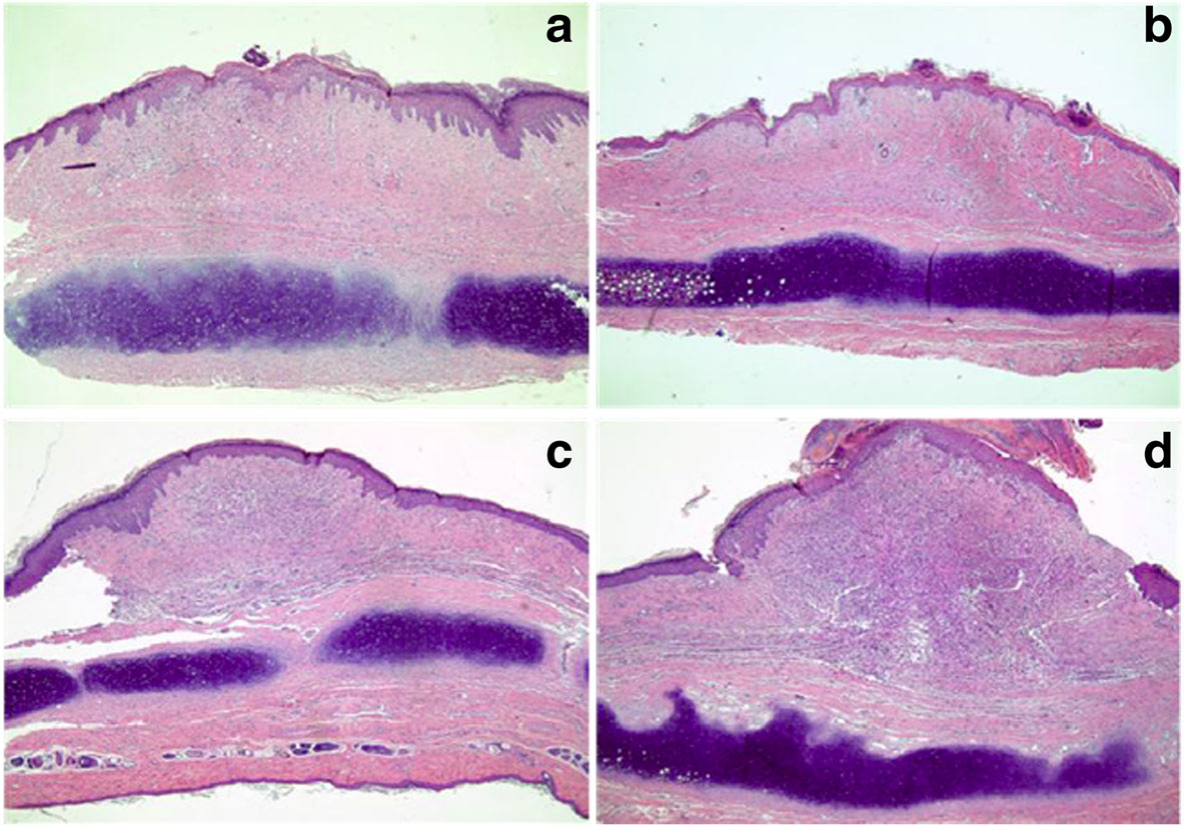
H&E histological analysis of rabbit hypertrophic scars after applied different treatments for 7 days. The 5EL group: the enthosomal gels encapsulated with 5-FU combined with CO_2_ fractional laser (**a**); CO_2_ group: CO_2_ fractional laser treatment (**b**); 5E group: the enthosomal gels encapsulated with 5-FU (**c**); and blank group: blank (**d**). Original magnifications: × 40

**Fig. 8:**
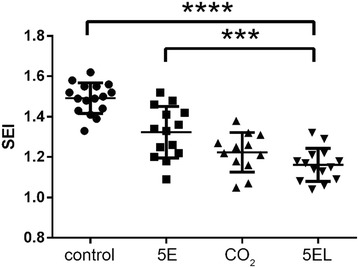
Comparison of SEI of rabbit hypertrophic scars after applied different treatments for 7 days. 5EL: the enthosomal gels encapsulated with 5-FU combined with CO_2_ fractional laser, *n* = 14; CO_2_: CO_2_ fractional laser, *n* = 12; 5E: the enthosomal gels encapsulated with 5-FU, *n* = 14; control: blank, *n* = 16. ***p value < 0.001 was considered statistically extremely significant and ****p value < 0.0001 was considered statistically highly extremely significant

We also compared the morphology of rabbit ear hypertrophic scar to determine the difference of using CO_2_ fractional laser. In the 5EL group, hypertrophic scars flattened and the color significantly turned to be light pink (Fig. 6a–b). In CO_2_ fractional laser treatment only group, hypertrophic scar thickness decreased to some extent and its color turned into a pale red (Fig. 6c–d). In the 5E group, hypertrophic scar thickness slightly decreased but the color remained bright red (Fig. 6e–f). Finally, there was no significant change in the untreated group compared with before treatment (Fig. 6g–h). Next, H&E staining was used for pathological analysis in 7 days after treatment for these four groups on rabbit ears hypertrophic scar tissue (Fig. 7). The 5EL group and CO_2_ fractional laser treatment only groups showed the decreased dermal layer thickness of hypertrophic scars and point-like crust with rare superficial dermis collagen fibers nodular or spiral-shaped arrangement (Fig. 7a–b). A large number of dermal collagen fiber penetration, disordered arrangement of collagen fibers, and superficial dermis nodular or spiral-shaped arrangement were found in the 5E group and untreated group (Fig. 7c–d). Another important evaluation method for hypertrophic scar is the scar elevation index (SEI). Similar with relative thickness pattern, SEI of 5EL group (1.16 ± 0.08) and CO_2_ fractional laser treatment only group (1.22 ± 0.10) was significantly decreased compared with the 5E group (1.32 ± 0.13) and untreated group (1.49 ± 0.08) (Fig. 8). Taken together, morphology analysis and SEI calculation data show CO_2_ fractional laser functions on healing rabbit ear hypertrophic scar in vivo.

## Discussion

Drug treatment is the main treatment approach, mainly topical or as local injections, for non-surgical treatment of hypertrophic scars. Because of the side effects of various drugs, local injection is often limited to small dosages and thus allowing only a small range of treatment. Also, due to the short half-life of drugs, persistent high concentration in scar require repeated injections [6, 30, 31]. In addition, because of the scar tissue is very concentrated and with severe pain during injections is often experienced, patients often do not accept for treatment. Although external drug usage having advantage of being convenient, painless, with long-term stability, low side effects, avoiding to affection of the gastrointestinal environment [7, 8], there are several limitations. First of all, topical and injected drugs availability is limited by special pathological scar tissue structure, which presents with a thickening of the stratum corneum and hyperplasia of the dermis, which hinders the penetration of the drug and achieving an effective therapeutic concentration. Recent reports have proven this and described how external use of drugs inefficiently penetrates the scar tissue [4, 9]. Ethosomes, proposed by Touitou et al. [10] in 2000 as a transdermal drug carrier, have been widely reported [11, 12, 32]. In this study, we improved the ethosomes preparation process to nano-level (particle size 70~90 nm). This change of a new nanoscale dimensions and the spatial conformation of the ethosomes allow them to penetrate into the scar tissue through a narrowly tightly connected cell gap not only due to the small size, but also by the similarity to the cell membrane of scar tissue cells. Based on such penetration mechanisms, ethosomes become a convenient transdermal drug carrier [14, 33, 34]. However, the anti-scar drug 5-FU encapsulated with ethosomes is mainly concentrated in the epidermis and dermis, which is not conducive to fibroblasts in deeper layers of the dermis. Therefore, our purpose is to explore a method that would allow to increase the penetration of drugs and would promote the uniform dispersion of drugs in scar tissue.

So far, commonly used methods to promote the penetration are divided into two categories: the promotion of chemical substances and physical methods to enhance permeability. The physical enhancement techniques include electroporation, iontophoresis, laser, microdermabrasion, microneedle, pressure, radiofrequency induction, and sonography [35]. There are many different types of lasers that have been shown to promote percutaneous administration, and ablative fractional lasers become the most popular laser in recent years for promoting drug penetration into the skin [20]. Ablative fractional laser can not only extremely reduce drug dosage, but also be conducive to drug penetration into deep skin and achieve high local concentrations to obtain a therapeutic effect. CO_2_ fractional laser, Erbium-doped Yttrium Aluminum Garnet (Er: YAG) fractional laser, and Erbium-doped Yttrium Scandiu Gallium Garnet (Er: YSGG) fractional laser can produce ablative zone or microporous channels on the surface of the skin. Compared with Er: YAG fractional laser (2940 nm wavelength) and Er: YSGG fractional laser (2790 nm wavelength), CO_2_ fractional laser (10,600 nm wavelength) has lower water absorption coefficient, larger thermal damage, and greater destruction effect of stratum corneum of the epidermis, which is more conducive to promote penetration effect [36].

In this study, 5-FU retention in the 5EL group (24.42 ± 4.37 μg / cm^2^) was significantly higher than in the 5E group (12.45 ± 1.64 μg/cm^2^) in scar tissue of 24 h treatment in vitro. Additionally, in Rho-labeled assay, 5EL group has shown higher fluorescence intensity than 5E group at 1-, 6-, and 24-h treatment time points. Image analysis showed that fluorescence can be found distributed in the gasification zone and surrounding dermal tissue matrix after 1-h CO_2_ laser treatment in the 5EL group, and the fluorescence in 5E group is only distributed in the epidermis. After 6- and 24-h treatment, diffuse fluorescence range is wider and fluorescence intensity is higher in the 5EL group than in the 5E group. CO_2_ fractional laser-induced gasification zone provided an effective way for drug penetration through the skin scar tissue, enlarge range of dermis penetration depth, and retention content in scar tissue. There are three different forms of thermal effect damage by CO_2_ fractional laser acting on the skin or scar tissue, from center to outliner, the gasification zone, thermal coagulation necrosis zone, and thermal denaturation zone [37]. Fluorescence data showed that Rho fluorescence was distributed from more concentrated gasification zone to diffused tissue, suggesting that there is no effect for permeation of drugs in thermal coagulation necrosis zone and thermal denaturation zone, which also confirmed the feasibility of CO_2_ fractional laser for the promotion of topical anti-scarring drug penetration. Together, CO_2_ fractional laser is conducive to more anti-scarring drugs 5-FU retention in scar tissue and achieve high drug concentration required to strengthen the anti-scarring effect.

The duration of CO_2_ fractional laser enhancing 5E was evaluated by CLSM on rabbit ear hypertrophic scar in vivo. The opening rates of microporous channels for drug permeation were 100% (0 h), 100% (6 h), 90.59% (12 h), and decreased to 15.58% (24 h) after CO_2_ fractional laser treatment of hypertrophic scar. Moreover, microporous channel opening rates dropped to zero on 3 and 7 days, and the drug can no longer penetrate into the skin through these channels. These results were consistent with that of epidermal re-epithelialization after ablative fractional laser irradiation, which is the skin wound around the keratinocytes to the wound defect migration and proliferation, covering the wound to form a complete layer of cells formed by the epidermis. When the dermal layer of skin was wound, the repair process will immediately start and quickly rebuild the skin barrier [38, 39]. The whole skin tissue trauma repair process can be divided into four continuous and overlapping steps: coagulation, inflammation, re-epithelialization, and remodeling [40]. Human skin after ablation of the fractional laser irradiation injury area (including the gasification area and coagulation necrosis area) complete epidermal re-epithelialization in 2 to 3 days and dermal remodeling for at least 4 weeks [41]. Thus, although the lesion in the dermis does not heal within 24 h after the CO_2_ laser treatment, the epidermis has completed the complex epithelization, including the formation of the stratum corneum, where the topical drug could not penetrate through channels. Taken together, epidermis, especially the stratum corneum, is still the main barrier for drug penetration through the skin.

In addition, our CLSM data showed both in in vitro human hypertrophic scar skin and in vivo rabbit ear hypertrophic scar skin, Rho-labeled 5E can penetrate the necrotic coagulation layer and the formation of crust on the surface after the CO_2_ fractional laser irradiation. It suggested that the skin under the crust but not the coagulation necrotic layer and crust tissue can impede the penetration of drug. Therefore, 24 h is the critical time-point for the effect of CO_2_ fractional laser on the penetration effect of hypertrophic scar in rabbits.

It has been reported that the clinical application of exfoliative fractional laser is an effective method to treat various skin diseases (such as solar keratosis, basal cell carcinoma, Bowen’s disease, etc.) [25–28]. However, the clinical efficacy of CO_2_ fractional laser in combination with drugs has not been explored in the treatment of hypertrophic scars [42, 43]; in particular, there are no reports of combined CO_2_ fractional laser (physical technique) with anti-scar drug nano-level ethosomes (chemical substances promoting scar penetration) for the treatment of hypertrophic scars. Using in vivo study, we performed a rabbit hypertrophic scar model for validation of CO_2_ fractional laser protocol. On the seventh day after intervention, the relative thickness of the four groups of hypertrophic scar was measured: experimental group (CO_2_ fractional laser combined with 5-FU EG): 1.27 ± 0.15 < control group A (CO_2_ fractional laser irradiation only): 1.35 ± 0.09 < control group B (EG encapsulated with 5-FU only): 1.52 ± 0.10 < control group C (Blank control): 1.61 ± 0.15. The results were consistent with the SEI (scar index) measured by H&E staining: the experimental group: 1.16 ± 0.08 < control group A: 1.22 ± 0.10 < control group B: 1.32 ± 0.13 < control group C: 1.49 ± 0.08. The difference between the experimental group and control group B, experimental group and control group was statistically significant. However, there was no statistically significant difference between the experimental group and control group A, which suggests that CO_2_ fractional laser had a leading role in the intervention of hypertrophic scars. This finding was mainly manifested in three aspects. First of all, CO_2_ fractional laser generated micro-channels for the promotion of scar drugs penetration. Secondly, CO_2_ fractional laser itself could help in hypertrophic scar tissue’s collagen remodeling. Thirdly, 5E itself could directly influence hypertrophic scars. From H&E staining data, we found that processes of collagen fiber bundle remodeling, from disordered, different-direction collagen fibers into a consistent, paralleled direction collagen beam take place. In the experimental group of rabbit ear hypertrophic scar, there was a significant reduction in scar thickness, but also color change of hypertrophic scar, from bright red to light red, which may be induced by vascular proliferation of the scar tissue.

The toxicity of the ethosomes should be a big concern in this study. Nevertheless, after reviewing literature, there are no results exhibiting the toxicity of ethosomes in vitro or in vivo study [44–46]. The permeability mechanism of ethosomes is mainly as follows: high concentration of ethosomes, the flexibility, and fluidity of ethanol liposome membrane, makes ethosomes deform in the process of transmission, and enhances the permeability in scar tissue [47].

Although the difference between the experiment group and control group A was not statistically significant, the relative thickness and SEI of the experimental group was smaller than that in the control group A. Both groups were treated with CO_2_ fractional laser, with or without 5E. This finding suggests that CO_2_ fractional laser may have the dominant role, which overtakes the minor effect of 5E drug in the final anti-scar effect.

## Conclusion

CO_2_ fractional laser can rapidly and significantly promote 5-fluorouracil encapsulated ethosomes’ permeability through hypertrophic scars in vitro. CO_2_ fractional laser is a potentially efficient method of promoting drug permeation in hypertrophic scars’ treatment. Our hypertrophic scar model (rabbit) showed that CO_2_ fractional laser combined with external-loaded 5-fluorouracil encapsulated ethosomes can effectively cure hypertrophic scars. Also, CO_2_ fractional laser itself can facilitate collagen remodeling in hypertrophic scar of rabbit ears. CO_2_ fractional laser can significantly promote the permeation of 5-fluorouracil encapsulated ethosomes, but the effect begins to relinquish 24 h after CO_2_ fractional laser irradiation, which indicates that 24 h is a critical period.
